# Apolipoprotein E (*APOE*) Allele Frequencies and Genotypic Distribution in Huambo, Angola

**DOI:** 10.3390/genes17080918

**Published:** 2026-08-03

**Authors:** Job Pakisi, Vicente Martín-Sánchez, Cruz S. Sebastião, Sergio Rodríguez Núñez, Celina Capingala-Pakisi, Victor Moreno, Enrique Bayón-Darkistade

**Affiliations:** 1 Faculdade de Medicina (FM), Universidade José Eduardo dos Santos (UJES), Huambo, Angola; jobpakisi19@gmail.com (J.P.); celinapakisi40@gmail.com (C.C.-P.); 2 Instituto de Biomedicina, Universidad León, 24071 León, Spain; srodrn@unileon.es (S.R.N.); jebayd@unileon.es (E.B.-D.); 3 Consortium for Biomedical Research in Epidemiology & Public Health (CIBER Epidemiología y Salud Pública-CIBERESP), 28029 Madrid, Spain; v.moreno@iconcologia.net; 4 Centro de Investigação em Saúde de Angola (CISA), Instituto Nacional de Investigação em Saúde (INIS), Luanda, Angola; cruzdossantos10@gmail.com; 5 Centro Nacional de Investigação Científica (CNIC), Luanda, Angola; 6 Global Health and Tropical Medicine, GHTM, Associate Laboratory in Translation and Innovation Towards Global Health, LA-REAL, Instituto de Higiene e Medicina Tropical, IHMT, Universidade NOVA de Lisboa, UNL, Rua da Junqueira 100, 1349-008 Lisboa, Portugal; 7 Catalan Institute of Oncology, Institut d’Investigacio Biomedica de Bellvitge, 08908 Barcelona, Spain; 8 Colorectal Cancer Group, ONCOBELL Program, Institut d’Investigació Biomèdica de Bellvitge (IDIBELL), L’Hospitalet de Llobregat, 08908 Barcelona, Spain; 9 Department of Clinical Sciences, Faculty of Medicine and Health Sciences and Universitat de Barcelona Institute of Complex Systems (UBICS), University of Barcelona (UB), L’Hospitalet de Llobregat, 08908 Barcelona, Spain

**Keywords:** *APOE*, allele frequency, Angola, non-communicable diseases

## Abstract

**Background/Objectives**: Non-communicable diseases (NCDs) are a leading cause of death globally, particularly in low- and middle-income countries undergoing rapid epidemiological transition. Apolipoprotein E (*APOE*) polymorphisms have been implicated in modulating risk for cardiovascular and neurodegenerative diseases. This study characterizes the distribution of *APOE* alleles and genotypes in an Angolan population sample and explores associations with demographic variables. **Methods**: A cross-sectional study was conducted in Huambo Province, Angola, with 200 unrelated adults aged 40–70 years. Genotyping for ε2, ε3, and ε4 was performed via real-time PCR. Allelic and genotypic frequencies were calculated and tested for Hardy–Weinberg equilibrium. Associations with sex, age (≤54 vs. >54 years), and urban vs. rural residence were analyzed. **Results**: ε3 was the most frequent allele (63%), followed by ε4 (24.8%) and ε2 (12.2%). The ε3 homozygous genotype was prevalent (44%), with ε3/ε4 (19%) and ε2/ε3 (18%) also common. Significant Hardy–Weinberg disequilibrium was observed due to excess ε4 homozygotes and deficit of certain heterozygotes; possible contributing factors, including population structuring, convenience sampling, and technical aspects of genotyping, are discussed. ε4 prevalence was higher in urban residents and older individuals, while ε3 and ε2 were more common in rural areas and younger participants. **Conclusions**: Our results reveal notable genetic heterogeneity and highlight the epidemiological and evolutionary importance of ε4 in Angola. The findings underscore the need for integrating population genomics into public health strategies targeting NCD prevention, especially in rapidly urbanizing African contexts.

## 1. Introduction

Non-communicable diseases (NCDs) account for three out of every four deaths worldwide, and three out of four of these deaths occur in low- and middle-income countries [1]. Demographic changes and lifestyle transitions underlie the high prevalence of these diseases and their concerning rapid increase in emerging countries [2]. Additionally, interactions between genetic and environmental factors play a crucial role in the incidence and course of these diseases and must be considered when designing prevention and control strategies [3,4].

Apolipoprotein E (*APOE*) contributes to the modulation of immune and inflammatory responses, and its different polymorphisms can have differential effects on some of the most relevant NCDs such as cardiovascular, autoimmune, and neurodegenerative diseases [5,6]. The clinical relevance of *APOE* alleles lies in their pleiotropic impact: the ε3 allele is usually associated with a balanced lipid profile, ε2 with some cardiovascular protection in heterozygotes (though with a risk of dyslipidemia in homozygotes), and ε4 with a pro-atherogenic phenotype characterized by increased LDL cholesterol, decreased HDL cholesterol, and a greater predisposition to both atherosclerosis and cognitive decline including Alzheimer’s disease [7].

The global distribution of *APOE* alleles shows marked geographic and ethnic gradients [8]. Worldwide, the ε3 allele, with prevalences exceeding 70%, is the most frequent and considered the wild-type allele. The ε4 allele shows variable frequency and can reach up to 30% in some African populations. The ε2 allele is the least common, generally not exceeding 10%. These differences reflect patterns of positive selection, environmental pressures, and human migrations [8].

Angola, a Southwestern African country characterized by remarkable ethnic heterogeneity and a history marked by migrations and admixture [9], constitutes a unique setting to analyze the genetic variability of *APOE* and its relationship with emerging chronic diseases, which cause one in four deaths in the country [10]. This study aims to characterize the frequencies of *APOE* polymorphisms in Angola and their relationship with demographic variables.

## 2. Materials and Methods

### 2.1. Study Design and Setting

A cross-sectional study was conducted in Huambo Province, Angola, including a convenience sample of 200 unrelated adults (100 men and 100 women), half residing in urban areas and half in rural areas. Participants were aged between 40 and 70 years (mean 55 ± 9 years; median 54). The population of Huambo Province is predominantly composed of the Bantu Ovimbundo ethnic group; the sample is therefore relatively ethnically homogeneous and is not intended to capture the full ethnic diversity of Angola. Urban participants were recruited in the municipality of Huambo through public institutions after an informational session and written informed consent; rural participants were recruited from among residents of several rural municipalities who were staying at a religious institution at the time of data collection. Exclusion criteria included major neurological diseases, known high risk of severe cardiovascular disease, pregnancy, and HIV infection. The study was approved by the Scientific Council of the Faculty of Medicine of Huambo (Deliberation No. 003/CC/23) and the Ethics Committee of the Angolan Ministry of Health (031/C.E.M.S./2023).

### 2.2. Sample Collection and Processing

Peripheral blood was collected in EDTA tubes. Genomic DNA was extracted using the QIAamp DNA Mini Kit (QIAgen, Hilden, Germany). *APOE* genotyping for alleles ε2, ε3, and ε4 was performed by real-time PCR (StepOne 7500, Applied Biosystems, Waltham, MA, USA) with SYBR Green and specific primers designed from the human APOE reference sequence (NG_007084.2, NCBI). Allele-specific discrimination was achieved with three primer combinations, each producing a 178-bp amplicon: the ε2 reaction (primers ApoE-112C and ApoE-158C), the ε3 reaction (primers ApoE-112C and ApoE-158R), and the ε4 reaction (primers ApoE-112R and ApoE-158R), corresponding to the two polymorphic sites that jointly define the ε2/ε3/ε4 alleles [10]. Each 20-µL PCR reaction contained 1× Power SYBR Green PCR Master Mix (Applied Biosystems, Waltham, MA, USA), 0.3 µM of each primer, and 2 µL of genomic DNA; cycling conditions were 95 °C for 10 min followed by 40 cycles of 95 °C for 15 s and 62 °C for 1 min (StepOne Real-Time PCR System, 96-well format), using the comparative Ct method. All reactions were run in duplicate together with no-template negative controls and a previously genotyped positive control; a Ct value < 25 indicated allele positivity, following the validated allele-specific PCR protocol of Calero et al. [10]. Demographic data collected included sex, age (categorized as ≤54 or >54 years), and place of residence (urban or rural). Allelic and genotypic frequencies were calculated from the PCR results [10].

### 2.3. Statistical Analysis

Descriptive statistics were used to calculate allelic and genotypic frequencies, expressed with 95% confidence intervals (Clopper–Pearson exact method) [11]. Hardy–Weinberg equilibrium was assessed with chi-square tests (α = 0.05). The inbreeding coefficient (F-IS) was computed as F-IS = (He − Ho)/He, where He is expected heterozygosity and Ho is observed heterozygosity [12]. Allelic frequencies were compared using contingency chi-square tests; genotypic frequencies were compared using Fisher’s exact test. Differences in sex and age-group distribution between urban and rural participants were assessed with the chi-square test, and mean age was compared with the Student’s *t*-test. To evaluate the joint associations of sex, age group (≤54 vs. >54 years), and residence (urban vs. rural) with APOE status, multivariable logistic regression models were fitted with ε4-carrier status (ε2/ε4, ε3/ε4, or ε4/ε4 vs. other genotypes), and separately, ε2-carrier status (ε2/ε2, ε2/ε3, or ε2/ε4 vs. other genotypes) as binary outcomes, with sex, age group, and residence entered simultaneously as covariates; results are reported as odds ratios (ORs) with 95% confidence intervals. A full multinomial model across all six genotypes was not pursued because the rarest genotype (ε2/ε4) comprised a single participant, which would produce unstable estimates; the carrier-status approach is standard in the APOE epidemiological literature and directly addresses the sex/age/residence associations of interest. All analyses were performed using Stata version 16 (StataCorp, College Station, TX, USA).

## 3. Results

**Figure 1:**
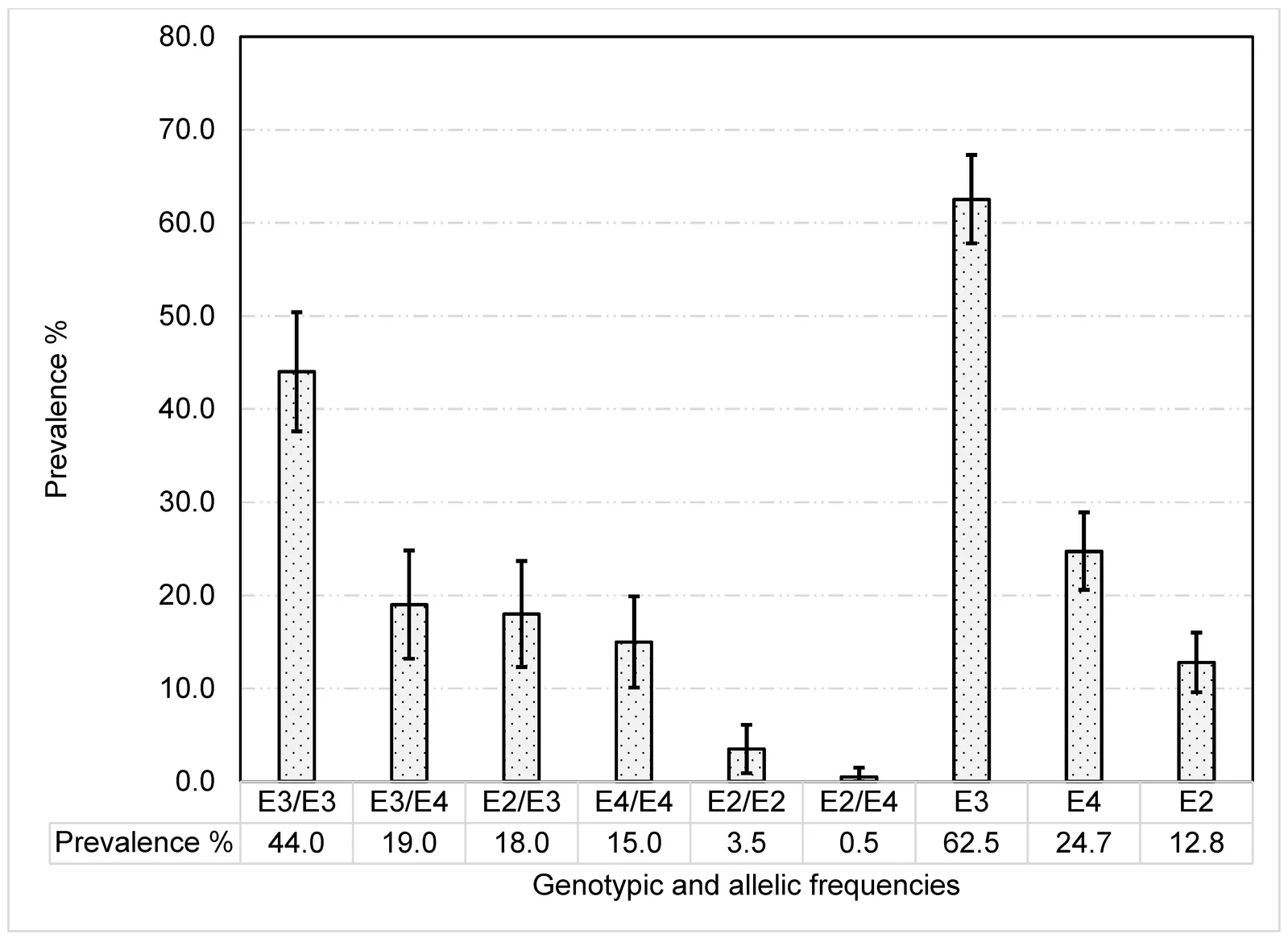
Distribution of genotypes and allelic frequencies and their 95% confidence intervals.

**Figure 2:**
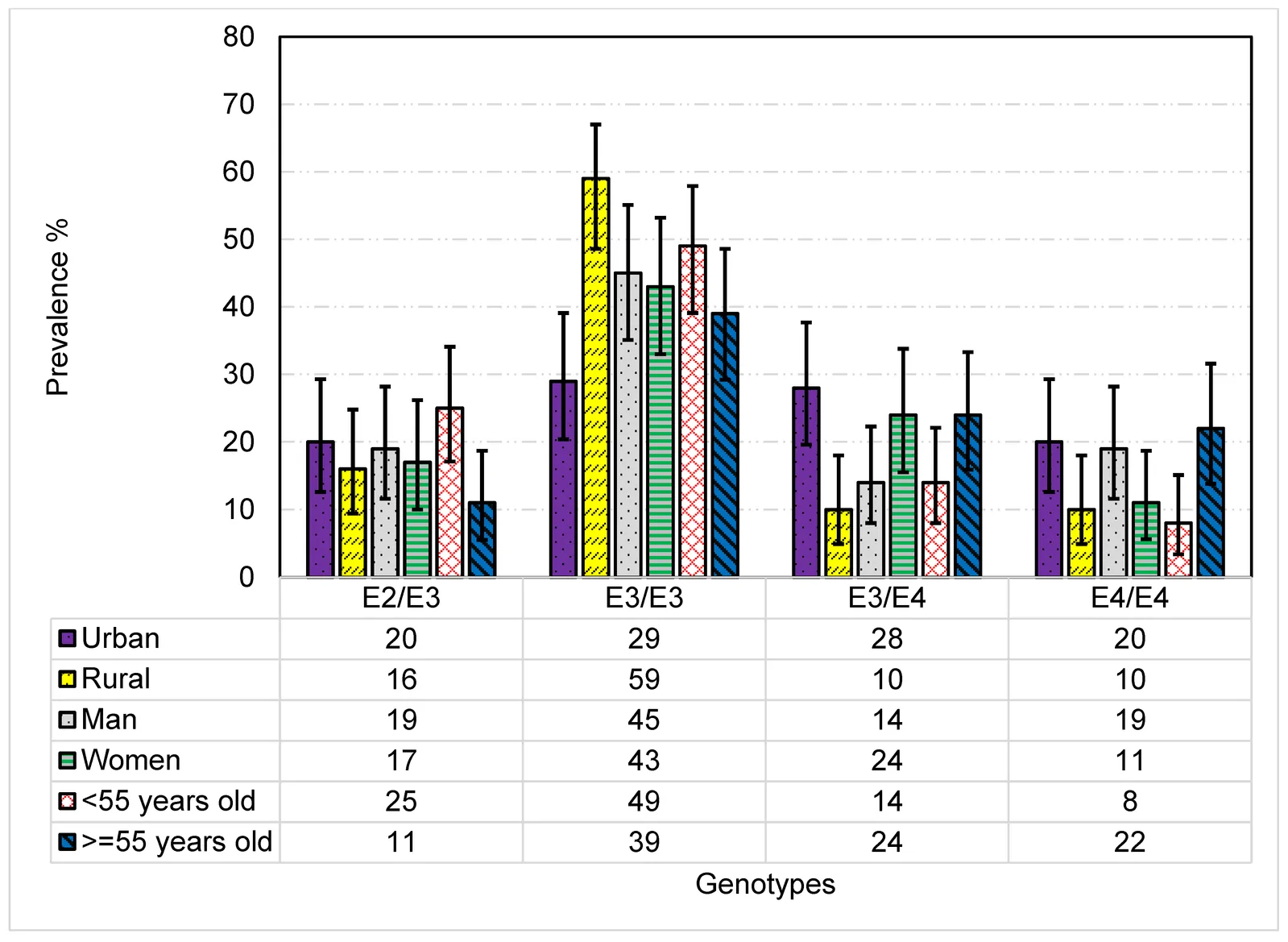
Distribution of genotypic variables according to the different categories analyzed.

The ε3 allele had the highest frequency, present in approximately two-thirds of participants, followed by ε4 (24.8%) and ε2 (12.2%) (Table 1, Figure 1). Genotypic analysis revealed 62.5% homozygotes and 37.5% heterozygotes. The most frequent genotype was ε3 homozygous (44.0%; 95% CI: 37.6–50.4), followed by ε3/ε4 (19.0%; 95% CI: 13.2–24.8), ε2/ε3 (18.0%; 95% CI: 12.3–23.7), and ε4/ε4 (15.0%; 95% CI: 10.1–19.9). ε2/ε2 and ε2/ε4 genotypes were the least frequent (3.5% and 0.5%, respectively). Hardy–Weinberg equilibrium testing showed significant deviation (χ^2^ = 51.73; df = 3; *p* < 0.001), driven by an excess of ε4 homozygotes and a deficit of ε2/ε4 and ε3/ε4 heterozygotes. The inbreeding coefficient (F_IS = 0.295) indicated a substantial deficiency of heterozygotes. This pattern persisted across sexes, urban/rural residence, and age groups. Significant differences were observed in allelic and genotypic distributions by residence and age but not sex. ε3 was more prevalent in rural areas (72.0% vs. 53.0% urban), while ε4 was more prevalent in urban residents (34.0% vs. 15.5% rural). Similarly, ε3/ε3 homozygotes predominated in rural (59.0%) versus urban (29.0%) areas, with ε3/ε4 heterozygotes more common in urban settings (28.0% vs. 10.0%). Age comparisons showed higher ε4 frequency in older participants (>54 years: 34.3% vs. ≤54 years: 15.4%) and higher ε2 in younger individuals (16.8% vs. 8.6%), with corresponding genotypic differences (ε2/ε3 and ε4/ε4). In Figure 2, the distribution of genotypic variables according to the different categories analyzed can be found.

Because the urban and rural subsamples were recruited through different channels (see Section 2.1), their comparability with respect to sex and age was formally tested (Table 2). Sex was perfectly balanced by design between residence groups (50.0% male in both urban and rural participants; χ^2^ = 0.00, *p* = 1.000). Age-group distribution, however, differed significantly by residence: participants >54 years were more frequent among urban residents (57.0%) than rural residents (42.0%) (χ^2^ = 3.92, *p* = 0.048), although the mean continuous age did not differ significantly between groups (55.3 ± 8.7 years urban vs. 53.1 ± 8.8 years rural; *t*-test *p* = 0.085). This age imbalance is potentially relevant, since age was independently associated with ε4 carriage in the multivariable model below, and is addressed further in the Discussion section.

To evaluate whether sex, age, and residence were independently associated with APOE status when considered simultaneously, multivariable logistic regression models were fitted for ε4-carrier status and ε2-carrier status (Table 3). ε4 carriage was independently associated with older age (>54 years vs. ≤54 years: OR = 2.77, 95% CI: 1.46–5.25, *p* = 0.002) and with urban residence (OR = 3.19, 95% CI: 1.69–6.04, *p* < 0.001), but not with sex (OR = 1.33, 95% CI: 0.70–2.50, *p* = 0.381). Notably, the association between urban residence and ε4 carriage remained statistically significant after adjusting for the age imbalance described above, indicating that residence contributes independently of age to ε4 carriage in this sample. ε2 carriage was independently associated with younger age (>54 years vs. ≤54 years: OR = 0.37, 95% CI: 0.18–0.76, *p* = 0.007) but not with sex (*p* = 0.743) or residence (*p* = 0.440).

## 4. Discussion

This is the first study to describe the allelic and genotypic prevalences of apolipoprotein E in the Angolan population. Our findings highlight the remarkable genetic and phenotypic heterogeneity present in the African continent, as well as the coexistence of historical adaptive advantages with emerging risks stemming from changes in lifestyle [13,14,15] The ε4 allele, which confers an advantage in environments characterized by high infectious burden and nutritional limitations, is significantly associated with adverse impacts in urbanized settings with Western lifestyles, marked sedentarism, and high-calorie diets [7,15]. This represents a critical challenge for public health strategies aimed at preventing and managing emerging diseases, particularly cardiovascular and neurodegenerative disorders.

In our sample, the ε3 allele was the most prevalent, with an approximate frequency of 63%, a figure that falls within the commonly reported range in global and regional studies, where ε3 typically varies from 55% to 90% across different human populations, showing closer similarity to prevalences reported for other sub-Saharan groups [8,16]. It should be noted that from an evolutionary standpoint, ε4 rather than ε3 is generally considered the ancestral allele at this locus, with ε3 and ε2 arising later through sequential mutation and subsequently rising to high frequency in most modern human populations [17]. The high frequency of ε3 observed here therefore reflects its status as the currently dominant, but not ancestral, allele, and is broadly consistent with frequencies reported for other African and Afro-descendant groups [8,16], supporting the internal consistency of our results.

The frequency of the ε4 allele shows considerable worldwide variation, ranging from 0% in some Indian populations to nearly 50% in certain Brazilian or African tribes [18]. In our case, the observed value of 24.8% represents an intermediate level within the African spectrum. While North African regions, such as Morocco and Tunisia, report low frequencies (<10%), other West African countries (such as Senegal: 3%) also exhibit considerably lower levels. In contrast, countries like Uganda (25%), Rwanda (24%), South Africa (25.4%), Nigeria (30%), Sudan (29%), and particularly Khoisan groups (37%) and Central African pygmies (41%) present high prevalences [13,19,20]. This finding underscores the genetic heterogeneity within the African continent and emphasizes the uniqueness of the Angolan population structure.

The high prevalence of the ε4 allele, especially in its homozygous form, is one plausible contributor to the significant genotype disequilibrium observed. However, several alternative and non-mutually exclusive explanations must be considered before attributing this deviation to a single biological cause. First, the convenience sampling strategy used here may have introduced selection bias, as participants were not randomly drawn from the source population. Second, undetected genotyping error is a known risk in *APOE* typing, since the ε2, ε3, and ε4 alleles are distinguished by only two single-nucleotide polymorphisms (rs429358 and rs7412), and misclassification can spuriously inflate apparent homozygote excess. Third, population stratification arising from Angola’s marked ethnic heterogeneity could generate a Wahlund-type effect that mimics inbreeding at the population level even in the absence of true consanguinity. Only after these technical and sampling explanations are reasonably excluded can population structure and endogamy, consistent with localized marital practices reported in other African settings [13,14,21], be considered a likely contributor to the elevated inbreeding coefficient observed. A formal comparison of our allele frequencies with publicly available reference datasets supports this cautious interpretation: our results are broadly consistent with the largest available reference for continental African ancestry: in the gnomAD database (v4.1.0, June 2025), individuals of African ancestry show allele frequencies of approximately 22% for ε4 and 11% for ε2, with the remainder corresponding to ε3 (≈67%) [22]. Our observed frequencies (ε4 = 24.8%; ε2 = 12.2%; ε3 = 62.5%) fall close to these continental African estimates, suggesting that the Huambo sample is broadly representative of the wider pattern described for African populations, even though Sub-Saharan Africa itself shows considerable inter-ethnic variation, as illustrated by the range reported across Sub-Saharan cohorts (ε4 from 3% in Senegal to over 40% in Central African Pygmy groups) [13,18,19,20]. A dedicated comparison against Angola-specific or Southern African 1000 Genomes Project subpopulations was not possible, as no such reference panel currently exists for Angola specifically; this is itself a gap in the literature that our study helps to address.

This dynamic balance of risks and benefits underscores the importance of a personalized approach in medicine and public health for countries undergoing epidemiological transition.

It is important to emphasize that the present study collected only genotypic and demographic data; no phenotypic, biochemical, or clinical measurements (e.g., lipid profile, cardiovascular events, cognitive assessment) were obtained from the participants. Consequently, any discussion of disease risk below draws on the established literature rather than on direct evidence from this cohort, and should be interpreted as hypothesis-generating rather than as a demonstrated finding in this population. The epidemiological relevance of the ε4 allele lies in its well-established association, reported elsewhere, with increased risk of cardiovascular and neurodegenerative diseases in Western lifestyle contexts. However, in Africa, phenotypic plasticity attributable to environmental, infectious, and behavioral factors has been reported [23,24,25]. As documented by Masemola et al. in South Africa, rural–urban transitions and lifestyle changes may precipitate future cardiovascular epidemics in ε4 carriers, emphasizing the urgency of genetic-environmental surveillance in Angola to anticipate disease burden evolution [15].

Analyzing the biomedical impact of the ε4 allele, several studies have confirmed its relationship with elevated total cholesterol, LDL-c concentrations, and reduced HDL-c, findings consistent with extensive international evidence [26,27,28,29]. This positions ε4 as a potential driver of the epidemiological transition in developing countries, accelerating the prevalence of non-communicable diseases (NCDs). On the other hand, the ε2 allele, while usually associated with a favorable lipid profile in heterozygotes, can predispose to severe dyslipidemias (type III) in the presence of factors such as diabetes and excessive alcohol intake, reinforcing the multifactorial nature of clinical risk [30,31,32].

Regarding urban–rural differences, the predominance of ε3 in rural areas (72%) contrasts with the higher frequency of ε4 in urban environments (34%), probably due to internal migrations, inequalities in healthcare access, and lifestyle variations. This pattern suggests that urban exposure may amplify cardiovascular and neurodegenerative risk for ε4 carriers, in line with the global findings [33]. It is worth clarifying that a higher frequency of ε4 among urban residents does not imply that the urban environment causes or selects for ε4; rather, the allele’s adverse metabolic effects are thought to be more likely to manifest phenotypically under an urban, Western-type lifestyle (sedentarism, energy-dense diets) while remaining comparatively silent under the more physically active, traditional lifestyle prevalent in rural areas. The higher observed frequency of ε4 in the urban subsample most plausibly reflects the demographic history of rural-to-urban migration in Huambo Province, in which lineages or ethnic groups with a naturally higher baseline ε4 frequency may be over-represented among urban residents, rather than any selective process favoring ε4 carriers who move to cities. Because our design is cross-sectional and convenience-based, we cannot rule out that this urban–rural contrast partly reflects recruitment bias (e.g., differential willingness to participate, or differences in the ethnic composition of the urban versus rural sampling sites) rather than a true population-level difference; distinguishing between these possibilities would require a randomly sampled, ethnically-stratified cohort with residential-history data, which was beyond the scope of this study. Notably, because urban participants in our sample were, on average, somewhat older than rural participants (Table 2), age is a plausible confounder of the urban–ε4 association; however, multivariable logistic regression showed that urban residence remained independently associated with ε4 carriage after adjusting for age and sex (OR = 3.19, 95% CI: 1.69–6.04; Table 3), indicating that age alone does not explain this pattern.

Age distribution is also relevant: the higher prevalence of ε4 among older individuals (>54 years) and predominance of ε2 in younger cohorts is a cross-sectional observation that may reflect cohort effects (e.g., generational differences in ancestry or admixture) rather than differential survival, and any inference regarding selection or mortality would require longitudinal data that this study did not collect. While ε4 has traditionally been linked to reduced longevity [34], Lane et al. demonstrated that its impact on mortality varies significantly according to ethnic and geographic context, potentially exhibiting neutral effects in African and Afro-descendant groups [21]. A more recent study by Vivian et al. found no association between ε4 genotypes and mortality in elderly individuals ≥80 years; however, classical cardiovascular factors such as smoking and diabetes increased mortality risk, whereas physical activity and elevated systolic blood pressure reduced it [35]. Therefore, it is essential to deepen longitudinal studies incorporating environmental and clinical variables to clarify these effects [36].

### Limitations

Several limitations should be considered when interpreting these findings. First, the sample size (n = 200) was relatively small for population genetics purposes and limits the precision of allele- and genotype-frequency estimates, as reflected in the width of several confidence intervals; results should therefore be interpreted as preliminary. Second, participants were recruited as a convenience sample rather than through random or stratified population sampling. The sample was drawn predominantly from the Bantu Ovimbundo ethnic group, the majority population of Huambo Province, and is therefore relatively ethnically homogeneous rather than representative of Angola’s full ethnic diversity; findings should not be generalized to other Angolan ethnic groups without further study. Urban participants were recruited through public institutions in the municipality of Huambo following informational sessions and written informed consent, whereas rural participants were recruited among residents of different rural municipalities who were staying at a single religious institution at the time of data collection; this difference in recruitment channel could itself introduce clustering or selection effects that are difficult to fully disentangle from genuine urban–rural biological differences. Third, no phenotypic, biochemical, or clinical data were collected, so associations with cardiovascular or neurodegenerative disease risk discussed above are drawn from the external literature and were not directly tested in this cohort. Fourth, the cross-sectional design could not distinguish cohort effects from selection or survival effects in the age-related comparisons. Fifth, the real-time PCR genotyping method, while previously validated elsewhere [10], was not accompanied in this study by an independent confirmatory method for a subset of samples; genotyping error therefore cannot be entirely excluded as a contributor to the observed Hardy–Weinberg disequilibrium. Finally, although multivariable logistic regression was used to assess the independent associations of sex, age, and residence with ε4- and ε2-carrier status (Table 3), the modest sample size limits statistical power to detect smaller effects or to test interactions between these covariates (e.g., whether the age-residence association with ε4 differs by sex); larger, multicenter studies are needed to confirm and extend these associations.

## 5. Conclusions

In conclusion, these findings emphasize the urgency of public health policies integrating population genomics as a tool for risk assessment and healthcare planning, especially in rapidly changing urban African contexts. Targeted preventive interventions should be prioritized, as recommended by Masemola et al., to mitigate the rise of NCDs in genetically susceptible groups and to test new personalized medicine strategies. Future research should expand sample sizes, incorporate additional genetic markers and clinical-metabolic variables, and conduct multicenter and trans-ethnic meta-analyses following models such as Marini et al., which enable a comprehensive understanding of genetic, environmental, and population health interactions. This study contributes to describing the *APOE* genomic profile in Angola, demonstrating the predominance of the ε3 allele and a comparatively high frequency of ε4, and highlighting population structuring as a plausible, though not the only possible, contributor to the Hardy–Weinberg disequilibrium observed. These findings, together with their potential public health implications for African populations, should be confirmed in larger, representative samples incorporating phenotypic data before firm conclusions are drawn.

## Figures and Tables

**Table 1:** Distribution of genotypic and allelic variants according to various variables.

Genotypes and Alleles	1. Urban $	2. Rural $	0. Male *	1. Female *	0. Age &	1. Age &	N	Total, % (95% CI)
ε2/ε2	3.0 (0.6–8.4)	4.0 (1.1–9.8)	3.0 (0.6–8.4)	4.0 (1.1–9.8)	4.0 (1.1–9.9)	3.0 (0.6–8.5)	7	3.5 (0.9–6.1)
ε2/ε3	20.0 (12.6–29.3)	16.0 (9.4–24.8)	19.0 (11.6–28.2)	17.0 (10.0–26.2)	25.0 (17.1–34.1)	11.0 (5.5–18.7)	36	18.0 (12.3–23.7)
ε2/ε4	0.0 (0.0–3.6)	1.0 (0.0–5.6)	0.0 (0.0–3.6)	1.0 (0.0–5.6)	1.0 (0.0–5.4)	0.0 (0.0–3.6)	1	0.5 (0.0–1.5)
ε3/ε3	29.0 (20.4–39.1)	59.0 (48.6–67.0)	45.0 (35.1–55.1)	43.0 (33.0–53.2)	49.0 (39.1–57.9)	39.0 (29.2–48.6)	88	44.0 (37.6–50.4)
ε3/ε4	28.0 (19.6–37.7)	10.0 (4.9–18.0)	14.0 (8.0–22.3)	24.0 (15.5–33.8)	14.0 (8.0–22.1)	24.0 (15.9–33.3)	38	19.0 (13.2–24.8)
ε4/ε4	20.0 (12.6–29.3)	10.0 (4.9–18.0)	19.0 (11.6–28.2)	11.0 (5.6–18.7)	8.0 (3.4–15.1)	22.0 (13.8–31.6)	30	15.0 (10.1–19.9)
ε2	13.0 (8.3–17.7)	12.5 (7.9–17.1)	12.5 (7.9–17.1)	13.0 (8.3–17.7)	16.8 (11.7–21.9)	8.6 (4.7–12.4)	51	12.8 (9.6–16.0)
ε3	53.0 (46.1–59.9)	72.0 (65.8–78.2)	61.5 (54.8–68.2)	63.5 (56.9–70.1)	67.8 (61.4–74.2)	57.1 (50.2–64.0)	250	62.5 (57.8–67.3)
ε4	34.0 (27.5–40.5)	15.5 (10.5–20.5)	26.0 (19.9–32.0)	23.5 (17.7–29.3)	15.4 (10.3–20.3)	34.3 (27.7–40.9)	99	24.7 (20.6–28.9)
Genotypes	$ *p* = 0.0001		* *p* = 0.2813		& *p* = 0.0025			
Alleles	$ *p* <0.0001		* *p* = 0.85		& *p* <0.0001			

Note: “$” stands for the comparison between rural vs urban areas; “*” stands for Sex comparison; “&” Stands for age comparison.

**Table 2:** Sex and age distribution of the urban and rural participants.

Characteristic	Urban (n = 100)	Rural (n = 100)	*p*-Value
Sex, n (%)			
Male	50 (50.0)	50 (50.0)	1.000 χ^2^
Female	50 (50.0)	50 (50.0)	
Age group, n (%)			
≤54 years	43 (43.0)	58 (58.0)	0.048 χ^2^
>54 years	57 (57.0)	42 (42.0)	
Age, years (mean ± SD)	55.3 ± 8.7	53.1 ± 8.8	0.085 *t*-test

**Table 3:** Multivariable logistic regression for ε4-carrier and ε2-carrier status (n = 200).

Outcome	Covariate	OR (95% CI)	*p*-Value
ε4 carrier vs. non-carrier	Female vs. Male	1.33 (0.70–2.50)	0.381
	Age > 54 vs. ≤54 y	2.77 (1.46–5.25)	0.002
	Urban vs. Rural	3.19 (1.69–6.04)	<0.001
ε2 carrier vs. non-carrier	Female vs. Male	0.89 (0.45–1.77)	0.743
	Age > 54 vs. ≤54 y	0.37 (0.18–0.76)	0.007
	Urban vs. Rural	1.31 (0.66–2.63)	0.440

OR: odds ratio; CI: confidence interval. Each outcome was modeled separately by multivariable logistic regression with sex, age group, and residence entered simultaneously. Reference categories: Male, ≤54 years, Rural.

## Data Availability

The data supporting this study’s findings are available on request from the corresponding author.
